# Prediction for the Total MRI Burden of Cerebral Small Vessel Disease With Retinal Microvascular Abnormalities in Ischemic Stroke/TIA Patients

**DOI:** 10.3389/fneur.2020.00268

**Published:** 2020-04-16

**Authors:** Liming Shu, Jiahui Liang, Weiquan Xun, Hong Yang, Tao Lu

**Affiliations:** ^1^Department of Neurology, The Seventh Affiliated Hospital, Sun Yat-Sen University, Shenzhen, China; ^2^Department of Neurology and Stroke Center, The First Affiliated Hospital, Sun Yat-Sen University, Guangzhou, China; ^3^Department of Neurology and Stroke Center, The Fourth Affiliated Hospital of Guangxi Medical University, Liuzhou, China

**Keywords:** cerebral small vessel disease, stroke, retinal microvascular abnormalities, magnetic resonance imaging, retinal photography

## Abstract

**Background and Purpose:** The association of retinal microvascular abnormalities with the total cerebral small vessel disease (cSVD) burden found on brain MRI has not been determined. In the present study, we examined whether the retinopathy score could predict the total cSVD burden in ischemic stroke/transient ischemic attack (TIA) patients. A simple practical diagnostic tool may help identify candidates for MRI screening.

**Methods:** We consecutively collected clinical data including retinal photography and cerebral MRI of ischemic stroke/TIA patients from August 2016 to August 2017 at our stroke center. The retinopathy score was assessed by the Keith-Wagener-Barker grading system for analyzing retinal microvascular abnormalities. To evaluate the total cSVD burden, the total cSVD score was assessed by awarding one point for the presence of each marker of cSVD on MRI. The clinical characteristics and retinopathy score were analyzed across patients for each total cSVD score. The association between the retinopathy score and the total cSVD score was analyzed.

**Results:** Among the 263 enrolled patients, the frequency of hypertension in patients with a total cSVD score of 2, 3, or 4 was higher than that in patients with a score of 0 (69.5, 71.7, and 89.2% vs. 45.2% respectively, all *P* < 0.05). The retinopathy score was related to the total cSVD score (*r* = 0.687, *P* < 0.001). Adjusted multivariate ordinal regression showed that the retinopathy score was independently correlated with the total cSVD score (odds ratio [OR], 4.18; 95% confidence interval [CI], 3.07–5.70) after adjustment for age, history of hypertension, previous stroke/TIA and current smoking. The c statistics were 0.30 (95% CI, 0.24–0.37; *P* < 0.05), 0.46 (95% CI, 0.39–0.53; *P* = 0.303), 0.79 (95% CI, 0.72–0.86; *P* < 0.001), and 0.81 (95% CI, 0.74–0.88; *P* < 0.001) for predicting the total cSVD score of 1, 2, 3, and 4 respectively.

**Conclusions:** These results suggest that retinal microvascular abnormalities have predictive value for severe total cSVD burden in ischemic stroke/TIA patients.

**Subject Terms**: ischemic stroke, transient ischemic attack, retinal vessels, magnetic resonance imaging.

## Introduction

Cerebral small vessel disease (cSVD) is a term commonly used to describe a syndrome comprising clinical, cognitive, neuroimaging and neuropathological changes (1). Vascular risk factors such as age and hypertension are known to contribute to the pathogenesis of cSVD (2). The markers illustrating cSVD on cerebral magnetic resonance imaging (MRI) include lacunar infarct (LI), white matter hyperintensities (WMHs), enlarged perivascular space (EPVS), and cerebral microbleeds (CMBs) (1). Given the simultaneous occurrence and the joint effects of these MRI markers, a total cSVD measurement might better evaluate the combined effect of neuropathological changes in cSVD (2). Recently, a total cSVD burden score was obtained by summing these four MRI markers and was used to investigate the total cSVD burden among lacunar stroke and hypertensive patients, which revealed that the total cSVD burden is correlated with increased blood pressure (3), impaired cognitive function (4) and recurrent stroke (5).

Retinal vessels and cerebral small vessels share similar embryological origin, physiological properties, and anatomical features (6). The retinal vasculature directly visualized by an ophthalmoscope or retinal photography correlates with microangiopathic processes in the brain and can be a proxy for cerebral small vessels (7). The Keith-Wagener-Barker grading system has been widely used to evaluate retinal microvascular abnormalities in populations of general communities (8), Alzheimer's disease patients (9), brain microvascular disease patients (10), hypertensive patients (11), and pre-hypertensive patients (11). Retinal microvascular diseases share some vascular risk factors, such as hypertension, with cSVD. The association between retinal microvascular abnormalities and cSVD was demonstrated in previous studies by investigating individual cSVD marker on MRI (12–14). However, whether the total cSVD burden is related to retinal microvascular abnormalities in ischemic stroke/transient ischemic attack (TIA) patients has not been determined yet. In this study, we aimed to investigate the association between retinal microvascular abnormalities and the total cSVD burden in ischemic stroke/TIA patients and hypothesized that retinal microvascular abnormalities constitute an independent predictor of cSVD.

## Materials and Methods

### Subjects

We consecutively collected data from ischemic stroke or TIA patients admitted to our stroke center from August 2016 to August 2017. The inclusion criteria were as follows: (1) patients with a definite diagnosis of acute ischemic stroke confirmed by diffusion-weighted imaging (DWI) of cerebral MRI or a definite diagnosis of TIA established based on the duration of symptoms and the absence of acute cerebral infarction on DWI; (2) age 18–90 years; (3) retinal photography and cerebral MRI could be obtained; and (4) the duration from symptom onset to admission was <30 days. Patients were excluded according to the following criteria: (1) a history of ocular surgery or primary eye diseases involving the retina and retinal vessels or hindered observation of the fundus, such as retinal choroid inflammatory diseases, cataracts, or posterior detachment of the vitreous; (2) potential brain diseases, such as arteriovenous malformation, intracranial tumor, cerebral venous thrombosis, hydrocephalus, encephaledema caused by malignant infarcts or other diseases, and intracranial hypertension; (3) clinically significant heart failure or liver or kidney function deficiency; or (4) cSVD resulting from metabolic disease, toxicity, infection, immunity, heredity, injury, or other non-vascular factors.

The following data were analyzed for each patient: (1) demographic data; (2) clinical data including the national institute of health stroke scale (NIHSS) on admission, NIHSS at discharge, modified rankin scale (mRS) at discharge, and delay between stroke onset and cSVD detection; (3) current or former smoking, alcohol consumption, hypertension, hyperhomocysteinemia, atrial fibrillation, diabetes mellitus, hypercholesterolemia, heart disease and cerebrovascular disease; and (4) retinal photography and cerebral MRI including T1-weighted imaging, T2-weighted imaging, fluid-attenuated inversion recovery, magnetic resonance angiography, DWI, and susceptibility-weighted imaging (3.0 T clinical MRI scanner, Siemens, Erlangen, Germany).

Participants underwent an assessment of cerebrovascular risk factors during the course of the study according to a previous study (15). Systolic blood pressure ≥140 mmHg, diastolic blood pressure ≥90 mmHg, or the combination of self-reported hypertension diagnosis and use of antihypertensive medications at the time of examination was identified as hypertension. Diabetes mellitus was present if one of the following conditions existed: (1) the person was taking oral antidiabetics drug or insulin; or (2) fasting plasma glucose was ≥7.0 mmol/L (≥126 mg/dL), and random blood glucose was ≥200 mg/dL (11.1 mmol/L), and glycosylated hemoglobin A1– C was ≥7.0%; or (3) self-reported history of physician diagnosed diabetes mellitus. Hypercholesterolemia was defined as total cholesterol ≥6.2 mmol/L, and/or the use of antidyslipidemic medication, or a self-reported history of physician-diagnosed hypercholesterolemia or self-reported of lipid-lowering medication. According to the National Survey on Drug Use and Health, alcohol excess was defined as more than one standard drink per day (0.355 L or 12 oz of beer, 0.118 L or 4 oz of wine, or 0.044 L or 1.5 oz of liquor or distilled spirits), current smoking was defined as smoking any tobacco cigarettes in the last 30 days.

The Ethical Committee of the First Affiliated Hospital of Sun Yat-Sen University reviewed and approved this study (No. 2017146).

### Analysis of Retinal Microvascular Abnormalities

A conventional fundus camera (KOWA nonmyd7, Kowa Company, Ltd., Japan) was used for retinal photography. Two experienced ophthalmologists independently reviewed all images and were blinded to clinical data and MRI findings. The findings were classified using the Keith-Wagener-Barker grading system (16) as follows: (1) grade I, slight or modest narrowing of the retinal arterioles, with an arteriovenous ratio of ≥1:2; (2) grade II, modest to severe narrowing of retinal arterioles with an arteriovenous ratio <1:2 or arteriovenous nicking; (3) grade III, soft exudates or flame-shaped hemorrhages; and (4) grade IV, bilateral optic nerve edema. To determine the retinopathy score, a grade of I on the Keith-Wagener-Barker grading system was scored as 1 point, a grade of II was scored as 2 points, etc.

### Analysis of the Total cSVD Burden

Two experienced neurologists independently reviewed all images and were blinded to the clinical data and retinal photography findings. The definition of the standard criteria for each marker was based on the international consensus (1). We used the total cSVD score consisting of all four MRI markers of cSVD to evaluate the total cSVD burden based on the recently developed scoring system (2–4). One point was given for the presence of each of the following markers: either irregular periventricular hyperintensities extending into the deep white matter (Fazekas score 3) and/or (early) confluent deep white matter hyperintensities (Fazekas score 2 or 3), and the term, WMH, in this study was defined by the above criteria; a LI located in the internal or external capsule, basal ganglia, thalamus, or brain stem with a diameter <20 mm and not compatible with the clinical manifestation; a deep CMB located in the internal or external capsule, basal ganglia or thalamus; moderate to extensive EPVS in the basal ganglia with a scale of 2 or 3, according to a 3-category ordinal scale (0–10; 10–25; >25), in the hemisphere with the highest number of EPVSs. Scores ranged from 0 to 4 and represented the severity of the total cSVD burden.

### Statistical Analysis

All statistical analyses were performed with IBM SPSS Statistics for Windows software (Version 22.0, IBM Corp., Armonk, NY, USA). Continuous and ranked variables are summarized as the medians and interquartile ranges. Categorical variables are summarized as numbers and percentages. The chi-square test, Fisher exact test and Kruskal-Wallis test were used to detect differences where appropriate. Bonferroni correction was used in the *post hoc* analysis. Spearman correlation analysis was used to evaluate the correlation between the retinopathy score and the total cSVD score. Multivariate ordinal regression was used to assess the association between the retinopathy score and the total cSVD burden after adjusting for age, sex, history of hypertension, history of stroke, and current smoking. The odds ratio (OR) and 95% confidence interval (CI) were obtained. The value of the retinopathy score for predicting the total cSVD was evaluated by receiver operating characteristic curve (ROC) analysis and c statistics. The results were considered statistically significant if the *P* < 0.05.

## Results

### Clinical Characteristics

A total of 263 patients were included in this study. Table 1 summarizes the demographic and clinical characteristics of all the included patients. The median age of the patients was 61 years (interquartile range [IQR], 51.0–70.0 years), and 72.2% of the patients were male. The medians of NIHSS on admission and at discharge were 5 (IQR, 2–6) and 2 (IQR, 1–4), respectively. The mRS at discharge was 0 (IQR, 0–1). The median delay from stroke onset to cSVD detection was 7 (IQR, 5–8) days. Vascular risk factors such as hypertension, current smoking, hyperlipidemia, and diabetic mellitus were frequent among all of the patients (65.8, 44.9, 42.2, and 33.8%, respectively), whereas fewer patients had other vascular risk factors, including alcohol consumption, previous stroke/TIA, atrial fibrillation, and hyperhomocysteinemia (15.6, 16.7, 7.2, and 4.9%, respectively). Only a small proportion of the patients were admitted due to a TIA (7.2%). The proportions of the patients with total cSVD scores of 0, 1, 2, 3, and 4 were 16.0, 23.6, 26.2, 20.2, and 14.1%, respectively. The clinical characteristics of patients with each cSVD marker are summarized in Supplementary Table 1.

**Table 1:** Clinical characteristics of included patients.

		**Total cSVD Score**				
	**All (*n* = 263)**	**0 *n* = 42)**	**1 (*n* = 62)**	**2 (*n* = 69)**	**3 (*n* = 53)**	**4 (*n* = 37)**
Age (Median, IQR)	61 (51.0–70.0)	46.5 (38.8–57)	59.5 (48.0–66.3)^*^	60.0 (53.0–71.0)^*^	67.0 (59.5–73.0)^*^	70.0 (60.5–81.0)^*^
Male, *n* (%)	190 (72.2)	29 (69.0)	44 (71.0)	52 (75.4)	41 (77.4)	24 (64.9)
NIHSS on admission (Median, IQR)	5 (2–6)	4.5 (3–6)	4 (2–6)	5 (2–6)	4 (3–6)	5 (2–6)
NIHSS at discharge (Median, IQR)	2 (1–4)	2.5 (1–4)	2 (1–3)	3 (1–5)	2 (1–5)	2 (1–4)
mRS at discharge (Median, IQR)	0 (0–1)	0 (0–1)	0 (0–1)	0 (0–2)	0 (0–2)	0 (0–1)
Delay from stroke onset to cSVD detection (Median, IQR)	7 (5–8)	7 (5–8)	7 (5–8.75)	7 (5–8)	6 (5–8)	7 (6–8)
Hypertension, *n* (%)	173 (65.8)	19 (45.2)	35 (56.5)	48 (69.5)^*^	38 (71.7)^*^	33 (89.2)^*^
Diabetes mellitus, *n* (%)	89 (33.8)	12 (28.6)	18 (29.0)	27 (39.1)	22 (41.5)	10 (27.0)
Hyperlipidemia, *n* (%)	111 (42.2)	22 (52.4)	24 (38.7)	31 (44.9)	21 (39.6)	13 (35.1)
Hyperhomocysteinemia, *n* (%)	13 (4.9)	3 (7.1)	2 (3.2)	4 (5.8)	2 (3.8)	2 (5.4)
Atrial Fibrillation, *n* (%)	19 (7.2)	2 (4.8)	7 (11.3)	4 (5.8)	2 (3.8)	4 (10.8)
History of stroke/TIA, *n* (%)	44 (16.7)	3 (7.1)	7 (11.3)	13 (18.8)	11 (20.8)	10 (27.0)
Alcohol consumption, *n* (%)	41 (15.6)	5 (11.9)	5 (8.1)	17 (24.6)	5 (15.1)	6 (16.2)
Current or former smoking, *n* (%)	118 (44.9)	20 (47.6)	24 (38.7)	40 (58.0)	25 (47.2)	9 (24.3)

*cSVD, cerebral small vessel disease; IQR, interquartile; NIHSS, national institute of health stroke scale; mRS, Modified Rankin Scale; TIA, transient ischemic attack*.

* *P < 0.05, compared with group cSVD Score 0*.

The analysis of the clinical characteristics among patients with different total cSVD scores showed a significant difference in age. Compared with the group with a total cSVD score of 0, the patients in the other four groups were significantly older. In addition, the frequencies of hypertension, previous stroke/TIA and current smoking were significantly different among the four groups of total cSVD scores. Hypertension was more frequent in the groups with total cSVD scores of 2, 3, and 4 than in the group with a total cSVD score of 0, whereas no significant differences were observed in the other clinical characteristics.

### Characteristics of cSVD

LI was the most frequently observed marker (77.6%) among the four markers of the total cSVD score (WMHs, CMBs, EPVS, and LI), whereas WMHs were the least frequently observed marker (25.9%). The frequencies of EPVS and CMBs were 47.9 and 41.8%, respectively (Supplementary Figure 1).

Among all enrolled patients, the major patterns of the total cSVD score were as follows: LI only (18.0%), LI + WMHs + EPVS + CMBs (14.0%), LI + EPVS (12.0%), LI + CMBs + EPVS (11.0%), and CMBs + LI (10.0%) (Supplementary Figure 1).

### Characteristics of Retinal Microvascular Abnormalities

Approximately half of the included patients suffered from Stage I (21.3%) or II (25.1%) retinopathy (Table 2). A proportion of the patients had Stage III or Stage IV retinopathy (6.8 and 1.1%, respectively). Stage I retinopathy was frequent in patients with a total cSVD score of 1 or 2 (27.4 and 37.7%, respectively). More than half of the patients with a total cSVD score of 3 or 4 had Stage II retinopathy (52.8 and 56.8%, respectively). Moreover, Stage III and IV retinopathy was found only in patients with a total cSVD score of 3 or 4. Approximately 45.7% of the included patients did not have significant retinopathy according to the Keith-Wagener-Barker grading system.

**Table 2:** The characteristics of retinal microvascular abnormalities of the patients with cSVD.

		**Total cSVD Score**				
	**All (*n* = 263)**	**0 (*n* = 42)**	**1 (*n* = 62)**	**2 (*n* = 69)**	**3 (*n* = 53)**	**4 (*n* = 37)**
**Retinal microvascular abnormalities**, ***n*** **(%)**						
Grade I	56 (21.3)	3 (7.1)	17 (27.4)	26 (37.7)	6 (11.3)	4 (10.8)
Grade II	66 (25.1)	0 (0)	3 (4.8)	14 (20.3)	28 (52.8)	21 (56.8)
Grade III	18 (6.8)	0 (0)	0 (0)	0 (0)	10 (18.9)	8 (21.6)
Grade IV	3 (1.1)	0 (0)	0 (0)	0 (0)	2 (3.8)	1 (2.7)
Median (IQR)	1.0 (0–2.0)	0 (0)	0 (0–1.0)	1.0 (0–1.0)^*^	2.0 (1.5–2.0)^*^	2.0 (2–2.5)^*^

*cSVD, cerebral small vessel disease; IQR, interquartile*.

* *P < 0.05, compared with group cSVD Score 0*.

**Figure 1:**
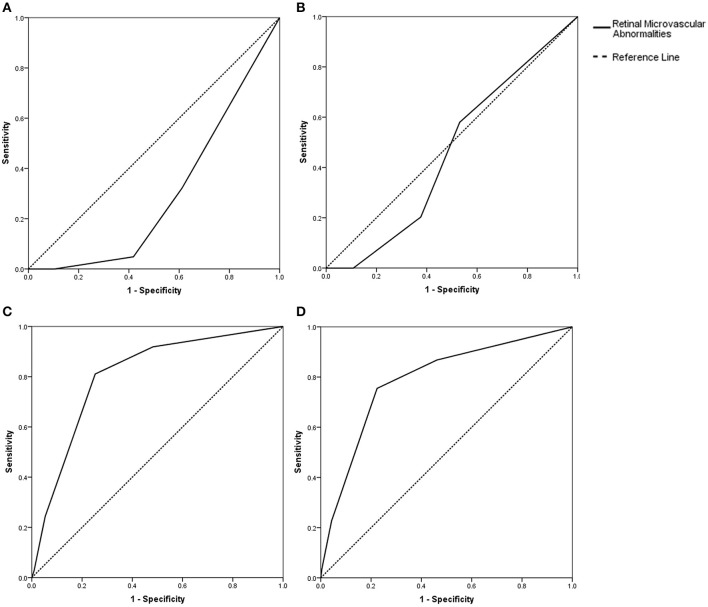
**(A)** Prediction with the retinopathy score (c statistics: 0.30, 95% CI, 0.24–0.37, *P* < 0.001) for the total cSVD score of 1. **(B)** Prediction with the retinopathy score (c statistics: 0.46, 95% CI, 0.39–0.53, *P* = 0.30) for the total cSVD score of 2. **(C)** Prediction with the retinopathy score (c statistics: 0.79, 95% CI, 0.72–0.86, *P* < 0.001) for the total cSVD of score 3. **(D)** Prediction with the retinopathy score (c statistics: 0.81, 95% CI, 0.74–0.88, *P* < 0.001) for the total cSVD score of 4.

A correlation analysis showed that the retinopathy score was related to the total cSVD score (*r* = 0.687, *P* < 0.001). Multivariate ordinal regression after adjustment for age, history of hypertension, previous stroke/TIA and current smoking, which is summarized in Table 3, showed that the retinopathy score (OR, 4.15; 95% CI, 3.05–5.65) was independently associated with the total cSVD score. The c statistics were 0.30 (95% CI, 0.24–0.37; *P* < 0.05, Figure 1A), 0.46 (95% CI, 0.39–0.53; *P* = 0.303, Figure 1B), 0.79 (95% CI, 0.72–0.86; *P* < 0.001, Figure 1C), and 0.81 (95% CI, 0.74–0.88; *P* < 0.001, Figure 1D) for predicting the total cSVD score of 1, 2, 3, and 4 respectively.

**Table 3:** Associations with total cSVD score and retinopathy score in multivariable ordinal regression analysis.

	**OR (95% CI)**
Retinopathy Score	4.18(3.07–5.70)^*^
Age	1.06 (1.04–1.08)^*^
Male	1.86 (1.01–3.41)^*^
Hypertension	1.07 (0.64–1.78)
Current or former smoking	1.09 (0.63–1.89)
History of stroke/TIA	2.46 (1.32–4.57)^*^

*OR, odds ratio; CI, confident intervals; TIA, transient ischemic attack*.

* *P < 0.05*.

## Discussion

In the present study, we found that the retinopathy score assessed by the Keith-Wagener-Barker grading system was independently associated with an increasing total cSVD score on MRI when adjusting for age, history of hypertension, previous stroke/TIA and current smoking in 263 ischemic stroke/TIA patients. The predictive value of the retinopathy score for a total cSVD score of 3 or 4 was statistically significant. These results suggest that retinal microvascular abnormalities have predictive value for a severe total cSVD burden.

Previous studies have focused only on individual cSVD markers instead of the total cSVD burden (3, 4, 17). Because MRI markers often occur simultaneously, a total cSVD measurement might better evaluate the combined effect of cSVD markers (2). The total cSVD measurement system proposed by Klarenbeek et al. (3) takes into account not only all four closely correlated MRI markers of cSVD but also the location, extent, and progression of each individual marker. Recent studies assessing the total cSVD score revealed that the total cSVD burden is correlated with age (11), an elevated blood pressure level (3) and an increased risk of recurrent stroke (5). In our study, we found that age and hypertension and previous stroke/TIA were significantly related to an increased total cSVD burden. Consistent with previous studies (3, 5, 11), our results suggest that the total cSVD score is a robust and reasonable measure for evaluating the total cSVD burden in ischemic stroke/TIA patients.

The retinal microvasculature shares common anatomical and physiological characteristics with cerebral arterioles (18). The structural and physiological characteristics of the blood-retinal barrier are analogous to those of the blood-brain barrier (6). Cerebral microangiopathy is thought to result from a breakdown of the blood-brain barrier (19), and retinal microangiopathy is caused by disruption of the blood-retinal barrier (20). Pathophysiological processes underlying retinopathy caused by hypertension, diabetes, and the other risk factors may mirror similar processes occurring in the brain and lead to the development of ischemic cSVD (8). Retinal microvascular abnormalities reflect cerebral arteriolar lesions induced by hypertension and other vascular risk factors (18). Retinal microvascular abnormalities such as focal arteriolar narrowing, arteriovenous nicking, soft exudates, flame-shaped hemorrhages, microaneurysms, and blot hemorrhages have been associated with an increased risk of stroke (7). Previous studies have demonstrated an association between retinal microvascular abnormalities and individual cSVD lesions including LI, WMHs, CMBs, and EPVS (7, 8, 21). A prospective study investigating US communities revealed that retinal microvascular abnormalities are independently associated with subsequent subclinical white matter lesions and infarctions after ~10.5 years of follow-up (8). The Rotterdam Scan Study found a correlation between the diameter of retinal venules and the severity of cSVD (21). Our data revealed that the retinopathy score assessed by the Keith-Wagener-Barker grading system was independently associated with the total cSVD score after adjusting for vascular risks such as age, history of hypertension, previous stroke/TIA and current smoking. Furthermore, we found a predictive value of retinal microvascular abnormalities for a severe total cSVD burden. cSVD is a major risk factor for stroke and dementia (22), which are considered a social and economic burden. Early identification and appropriate management of cSVD may be beneficial. A simple, practical diagnostic tool may help identify candidates for MRI screening and clinical follow-up. Retinal photography is routinely performed and sufficient for evaluating retinal abnormalities (7) and can be utilized to evaluate the microvasculature of patients at risk of cerebrovascular diseases (23). In China, the stroke care is improving in the recent years, but the stroke burden also keeps growing (24). A recent study focusing on the distribution of MRI scanners in China found the relative number of MRI scanners was 2.07 to 5.53 per million population, which was lower than the countries of Organization for Economic Co-operation and Development (25). Hence, combined with our findings, assessing retinal microvascular abnormalities may be a convenient method for evaluating the severity of total cSVD burden as well as clinical follow-up among ischemic stroke/TIA patients particularly in remote rural areas or hospitals without magnetic resonance equipment.

Previous studies on the association of the individual cSVD marker and the retinopathy are based on both the Asian cohort (14) and non-Asian cohorts (8, 26). Considering that cSVD is more common in Asian population than in other parts of the world (27) and the subjects in this study are all Asian, the association between the total cSVD burden and retinopathy maybe specific in Asian cohorts and should be further investigated in non-Asian cohorts.

This study focused on the relationship between total cSVD burden and retinal vasculature lesions in patients with ischemic stroke and TIA. The impact of recent infarct lesions on total cSVD burden is not clear, and the patients with TIA did not have infarct lesions. Therefore, the locations and the volumes of infarct lesions were not in the scope of this study and hence not analyzed in this manuscript. The association of recent infarct lesions and total cSVD burden can be explored in future studies.

In this study, we included the patients who were able to cooperate with retinal photography. Furthermore, the patients with encephaledema caused by malignant infarcts were excluded from this study. As a result, most of cases in this study were non-disabled stroke (the median mRS at discharge was 0). Hence, the patients of severe stroke were not included in this study. The patients with cSVD resulting from metabolic disease, toxicity, infection, immunity, heredity, injury, or other non-vascular factors were also excluded from this study. Thus, the association between total cSVD burden and retinopathy in the patients with non-vascular factors associated cSVD should be further explored in the future studies.

Our study has several limitations. First, we only included ischemic stroke/TIA patients who could undergo MRI scans and retinal photography. Consequently, patients with severe stroke were excluded. Thus, the severity of the total cSVD burden and retinal microvascular abnormalities may be underestimated. Second, in our study, the retinopathy score was a semi-quantitative method and might miss some subtle abnormalities of retinal vasculature that can be detected by quantitative assessments used in previous studies (12, 13, 21).

In conclusion, retinal microvascular abnormalities are independently associated with the severity of the total cSVD burden. These findings suggest that assessing retinal microvascular abnormalities might be helpful for evaluating the severity of total cSVD burden of ischemic stroke/TIA patients. And retinal photography is probably a convenient tool for evaluating cSVD.

## Data Availability Statement

The raw data supporting the conclusions of this article will be made available by the authors, without undue reservation, to any qualified researcher.

## Ethics Statement

The studies involving human participants were reviewed and approved by the Ethical Committee of the First Affiliated Hospital of Sun Yat-Sen University. Patients/participants provided their written informed consent to participate in the study.

## Author Contributions

TL, JL, and LS designed the study. TL obtained the data for the work. JL analyzed the data. TL and JL interpreted the data and drafted the study. HY provided advice for designing and drafting the work. TL revised all data and critically revised the paper for important intellectual content. All authors approved the final version of the paper to be published.

### Conflict of Interest

The authors declare that the research was conducted in the absence of any commercial or financial relationships that could be construed as a potential conflict of interest.
