# Supplementary material for: Prediction for the Total MRI Burden of Cerebral Small Vessel Disease With Retinal Microvascular Abnormalities in Ischemic Stroke/TIA Patients
Source: Front Neurol. 2020 Apr 16;11:268. doi: 10.3389/fneur.2020.00268 (PMC7177024; doi:10.3389/fneur.2020.00268)
Supplement: Supplementary file 1 [file Data_Sheet_1.PDF]

ONLINE SUPPLEMENT

Prediction for the Total MRI Burden of Cerebral Small Vessel Disease with Retinal Microvascular Abnormalities in Ischemic Stroke/TIA Patients - - online supplement

Table I. Clinical features of patients with different SVD

	LI（n=204）	CMBs（n=110）	EPVS（n=126）	WMH（n=68）
Age, Median (Interquartile)	63.5 (54~73)	65.0 (56~74.5)	65.0 (56.8~73.3)	71.0 (63~78)
Male, n (%)	147 (72.1)	84 (76.4)	91 (72.2)	45 (66.2)
Hypertension, n (%)	144 (70.6)	80 (72.7)	101 (80.2)	52 (76.5)
Diabetes mellitus, n (%)	72 (35.3)	34 (30.9)	53 (42.1)	20 (29.4)
Hyperlipidemia, n (%)	81 (39.7)	45 (40.9)	53 (42.1)	22 (32.4)
Hyperhomocysteinemia, n (%)	10 (4.9)	6 (5.5)	4 (3.2)	4 (5.9)
Atrial Fibrillation, n (%)	15 (7.4)	6 (5.5)	10 (7.9)	6 (8.8)
Previous Stroke/TIA, n (%)	39 (19.1)	21 (19.1)	29 (23.0)	17 (25.0)
Current Smoking, n (%)	91 (44.6)	48 (43.6)	53 (42.1)	23 (33.8)
Alcoho Consumption, n (%)	32 (15.7)	19 (17.3)	25 (19.8)	11 (16.2)
TIA, n (%)	11 (5.4)	4 (3.6)	10 (7.9)	1 (1.5)
The Keith–Wagener–Barker Grades, median (Interquartile)	1.0 (0~2.0)	2.0 (1.0~2.0)	2.0 (1.0~2.0)	2.0 (1.0~2.0)

cSVD, cerebral small vessel disease; WMHs, white matter hyperintensities; CMBs, cerebral microbleeds; EPVS, enlarge perivascular spaces; LI, lacunar infarcts.

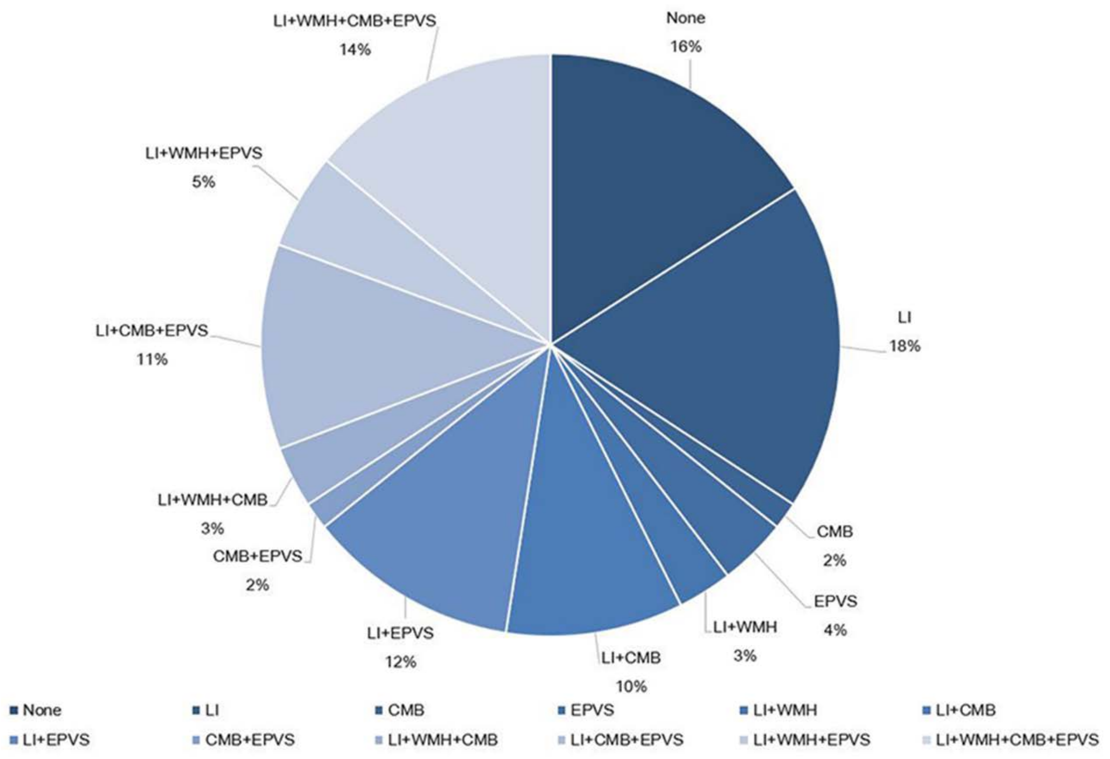

Figure I. The Frequencies of cSVD Markers. Due to all cSVD markers was seen in the patents with total cSVD score 4, the data of the combination was not shown in this figure. WMHs, white matter hyperintensities; CMBs, cerebral microbleeds; EPVS, enlarge perivascular spaces; LI, lacunar infarcts. In all the included patients, there were several patterns of total cSVD score markers including 1) LI only, 2) CMBs only, 3) EPVS only, 4) WMHs+LI, 5) CMBs+LI, 6) LI+EPVS, 7) LI+WMHs+CMBs , 8) LI+WMHs+EPVS , 9) LI+WMHs+EPVS, 10) LI+CMBs+EPVS , 11) LI+WMHs+EPVS+CMBs.
